# *Phoenix dactylifera* Protects against Doxorubicin-Induced Cardiotoxicity and Nephrotoxicity

**DOI:** 10.1155/2019/7395239

**Published:** 2019-12-23

**Authors:** Yuewen Wang, Xu Chao, Fiaz ud Din Ahmad, Hailong Shi, Hania Mehboob, Waseem Hassan

**Affiliations:** ^1^School of Basic Medical Sciences, Shaanxi University of Chinese Medicine, Xianyang, Shaanxi 712046, China; ^2^Department of Pharmacy, The Islamia University of Bahawalpur, Bahawalpur 63100, Pakistan; ^3^Department of Pharmacy, COMSATS University Islamabad, Lahore Campus, Lahore 54000, Pakistan

## Abstract

Doxorubicin (DOX) is an important anticancer drug used widely in the treatment of leukemia and lymphoma. The suitability of DOX is enhanced by its high therapeutic index, but its potential to cause cardiotoxicity and nephrotoxicity remains a prime concern in anticancer therapeutics. This study is designed to determine the effect of *Phoenix dactylifera* extract (PDE) on DOX-induced cardiotoxicity and nephrotoxicity. Experimental rats were divided into four groups, receiving normal saline 4 ml/kg, DOX alone, and crude extract of PDE at doses of 1 g/kg and 1.5 g/kg in the presence of DOX, respectively, for 21 days. Cardiac enzymes and serum and urinary sodium and potassium levels were evaluated which were analyzed statistically by using one-way ANOVA. Subsequently, DOX initiated changes in the level of cardiac markers CK-MB, LDH, and troponin I, which were notably reversed by PDE. PDE was also effective against serum and urinary sodium and urinary potassium and protected against DOX-induced nephrotoxicity. Groups treated with different doses of PDE showed marked decrease in levels of cardiac and renal markers. The study concluded that the PDE extract possesses protective effects against DOX-induced cardiotoxicity and nephrotoxicity.

## 1. Introduction

Doxorubicin (DOX) belongs to class of anthracyclines isolated from bacteria *Streptomyces peucetius* in the early 1960s. It is generally used in the treatment of leukemia, lymphoma, and solid cancers [1]. It is one of the main stay in anticancer therapy, and doxorubicin hydrochloride (HCl) liposomal injection was the first liposomal-encapsulated anticancer drug to receive clinical approval [1]. It was established in 1967 that its predecessor daunorubicin produces severe cardiac toxicity, which led to the genetic modification of *Streptomyces* spp. to produce adriamycin, which later came to be known as “doxorubicin” [2]. DOX not only kills the cancerous cells but also affects the normal cells of the body. It causes irreversible and dose-dependent cardiomyopathy and nephrotoxicity and hepatotoxicity. Although it has a higher therapeutic index, its cardiotoxic and nephrotoxic side effects still continue to exist [3]. DOX induces several structural and functional changes in cardiac tissues as the phospholipids in the mitochondrial membrane has a high affinity for DOX that accumulates it in heart cells [4]. DOX-induced cardiotoxicity occurs in three phases. The acute form arises in less than 1% patients immediately after infusion which is reversible if therapy is discontinued [5]. The subacute form appears within a week [6]. The chronic or late toxicity transpires within a year during DOX therapy which causes decreased left ventricle function and heart failure [7]. Pathogenesis of DOX-induced cardiomyopathy is implicated by multiple mechanisms including oxidative stress, downregulation of functional cardiac muscle-specific and mitochondrial proteins, inhibition and activation of enzymatic pathways, and exaggerated immune response. All these pathological events lead to altered molecular signaling and activation of apoptotic cascade resulting in the destruction of nucleic acid, sarcomere disruption, and myofibril loss [8]. However, the elevated mitochondria-to-cardiomyocyte ratio makes cardiomyocytes more susceptible to oxidative stress and has been proposed a key factor in pathogenesis compared with the aforementioned mechanisms. It has been proposed that DOX-semiquinone, an unstable metabolite of DOX, reacts with O_2_, producing H_2_O_2_ and O_2_^−^ (superoxide). In addition, DOX enhances the activity of extramitochondrial oxidative enzymes such as xanthine oxidase and NADPH oxidase and also interferes with mitochondrial iron export, resulting in formation of ROS (reactive oxygen species). On the contrary, DOX inhibits the activities of endogenous enzymatic and nonenzymatic antioxidants. So, an imbalance between ROS generation and neutralization leads to oxidative stress and has a greater damaging effect on heart compared with other organs such as the kidney and liver [8–10].

*Phoenix dactylifera* L. (family: Arecaceae) commonly known as date palm tree is a common staple for centuries around the globe. This ancient tree is indigenous to Arabia, Mexico, Australia, South America, USA, and North Africa [11, 12]. Small yellow color of flowers directly yields edible fruit in ready form which varies in shape, size, and weight [13]. Dates are rich in carbohydrates, proteins, fat, minerals, vitamins, dietary fibers, and vitamin B complex [14]. Historically, various tribes and traditional societies have used it for therapeutic purposes for centuries apart from its edible delicacies. The rise of scientific age has rationalized the already effective medicinal plant with abundant body of knowledge reported all around the globe [15]. Wide range of minerals, vitamins, and vital rich phytochemical constituents profile makes dates an effective candidate for diseases such as cancer, diabetes, and cardiovascular diseases [15]. Date fruit is reported to contain carotenoids, polyphenols, tannins, and sterols [16]. Arguably, date fruit contains the highest concentration of polyphenols among the dry fruits, predominantly due to greater sunlight exposure [17]. Polyphenols possess great antioxidant activity useful in cancers, cardiovascular disorders, and diabetes.

The effectiveness of date palm tree fruit has been reported in various diseases. Hepatotoxicity induced by various chemicals was reversed by *Phoenix dactylifera* [18, 19]. It is also found to be effective in diabetes [20], male infertility (seeds) [21, 22], inflammation, and bacterial infection [23]. Moreover, cardioprotective [24] and antioxidant actions [25] are also reported widely. However, the most attention-grabbing effects are highlighted for its activities against various kinds of cancers. Its anticancerous, antiproliferative, and antiangiogenic activities are reported against mammary cancer [11], colon cancer, [26] and breast cancers [27].

In view of its anticancer, antioxidant effects and effects against various toxicities, we attempted to examine the effects of date palm fruit extracts on DOX-induced cardiotoxicity and nephrotoxicity, which is one of the prevalent problems in the clinical use of DOX.

## 2. Materials and Methods

### 2.1. Preparation of Plant Extract

*Phoenix dactylifera* fruit was crushed in an electric pestle and mortar, grinded, and soaked in 70% methanolic solution for three days. Then, it was filtered, and the filtrate was then evaporated in a rotary evaporator at 30–40°C. The obtained semisolid residue was then kept in a refrigerator till further analysis.

### 2.2. Animals

Wistar albino rats, weighing 250–300 g, were kept in the experimental research laboratory, in the Islamia University of Bahawalpur, under 12 h light/dark cycle. The standard humidity (45–65%) and temperature (22–24°C) conditions were maintained. All the mice were provided with water and standard pallet diet ad libitum. Approvals of all the experimental protocols were taken from the Ethical Review Committee, Islamia University of Bahawalpur.

### 2.3. Experimental Design

Rats were randomly divided into four groups containing six animals each. Group-I was kept untreated and received normal saline via oral route for 21 days. Group-II was considered intoxicated and administered only with DOX at 10 mg/kg i.p. on day 1. Group-III was administered with DOX at a dose of 10 mg/kg and given PDE orally at a dose of 1 g/kg. Group-IV was also intoxicated with DOX at a dose of 10 mg/kg and coadministered with 1.5 g/kg orally.

### 2.4. Measurement of Cardiac Enzymes

Cardiac enzymes (CK-MB, LDH, and troponin I) are measured by using the ELISA kit. Elisa kits were purchased from Thermo Fischer. All the tests were performed according to the manufacturer's instruction.

### 2.5. Measurement of Serum and Urinary Sodium and Potassium

For the measurement of sodium and potassium levels in all the experimental groups, urine was poised on 0, 7^th^, 14^th^, and 21^st^ day of the study. Rats were kept in metabolic cages for 24 hours with free access to tap water. Water intake and urine output was measured regularly. For the estimation of sodium and potassium levels, samples were kept at −30°C. A flame photometer (Sherwood model 410, UK) was used to measure the amount of sodium and potassium in plasma and urine samples. For the measurement of sodium in plasma, samples were diluted as 1 : 200, and the same dilution was used for the measurement of potassium. Identical dilution was made to estimate potassium levels in urine, but for the sodium level, the 1 : 1000 dilution was used. The changes in urinary sodium-to-potassium ratio were measured on three different durations during the study period starting with day 0, continuing on day 10, and finally on day 21.

### 2.6. Histopathology

A section of heart and kidneys were fixed in 10% V/V neutral-buffered formalin and then processed for dehydration by passing them through pools of ethanol having different concentrations. Then, paraffin blocks were prepared, and 5 *μ*m thick sections were cut for staining with hematoxylin and eosin (H&E).

### 2.7. Statistical Analysis

Statistical analyses were conducted using GraphPad Prism version 5 software and by applying one-way ANOVA followed by Bonferroni post hoc test. The data were represented with mean ± standard deviation (SD). The *p* value ≤0.05 was considered statistically significant.

## 3. Results

### 3.1. Cardioprotective Effects of PDE

#### 3.1.1. PDE Ameliorates Elevated Cardiac Enzymes

**Figure 1:**
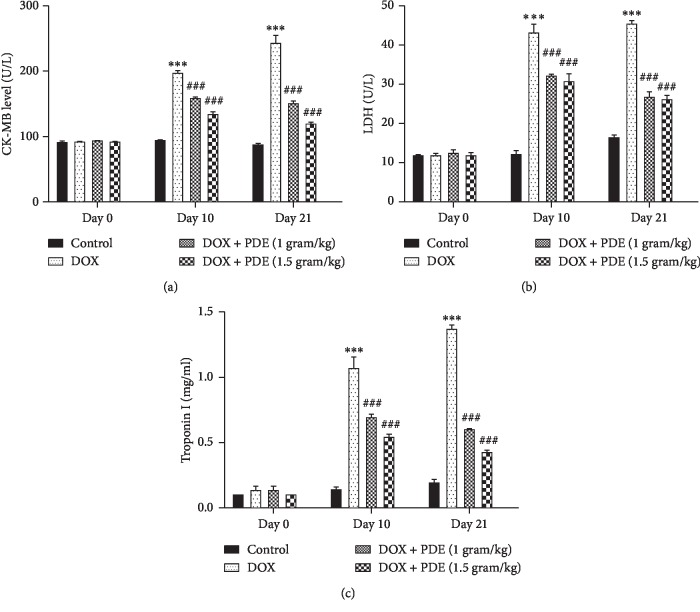
Effects of PDE on serum (a) CK-MB, (b) LDH, and (c) troponin levels in DOX-induced changes in rats. The values are mean ± SEM (*n* = 6). Statistical analysis was done through two-way analysis of variance (ANOVA) followed by Bonferroni post hoc test. For determination of *p* values, DOX is compared with the control group, and treatment groups were compared with the DOX group. The results are considered significant if *p* < 0.05. ^*∗∗∗*^*p* < 0.001 vs. control and ^###^*p* < 0.001 vs. DOX.

Elevated levels of serum concentration of different cardiac biomarkers such as CK-MB (Figure 1(a)), LDH (Figure 1(b)), and troponin I (Figure 1(c)) showed quantitative index of myocardial damage induced by DOX compared with the normal control group, and values were markedly lowered in groups treated with DOX.

#### 3.1.2. Effect of PDE on Serum Sodium Level

**Figure 2:**
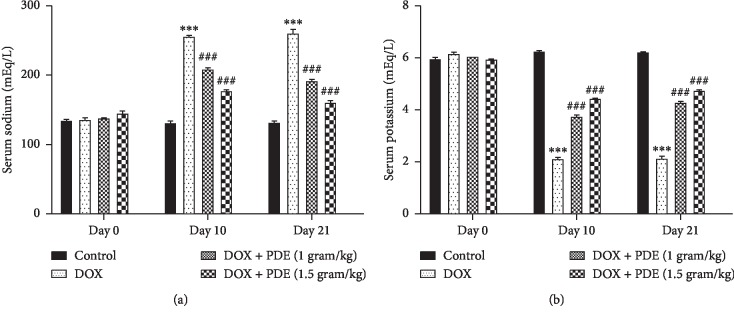
Effects of PDE on serum (a) sodium and (b) potassium levels in DOX-induced changes in rats. The values are mean ± SEM (*n* = 6). Statistical analysis was done through two-way analysis of variance (ANOVA) followed by Bonferroni post hoc test. For determination of *p* values, DOX is compared with the control group, and treatment groups were compared with the DOX group. The results are considered significant if *p* < 0.05. ^*∗∗∗*^*p* < 0.001 vs. control and ^###^*p* < 0.001 vs. DOX.

Level of serum sodium was increased in group treated with DOX at a single dose of 10 mg/kg compared with the normal control group, while there is notable reduction in sodium level in groups treated with PDE at a dose of 1 g/kg and 1.5 g/kg (Figure 2(a)). On the contrary, serum potassium levels (Figure 2(b)) are decreased in the DOX-induced group. Serum potassium levels were significantly brought back near normal by PDE both dose and time dependently.

### 3.2. Nephroprotective Effects of PDE

#### 3.2.1. Effect of PDE on Urine Flow Rate

**Figure 3:**
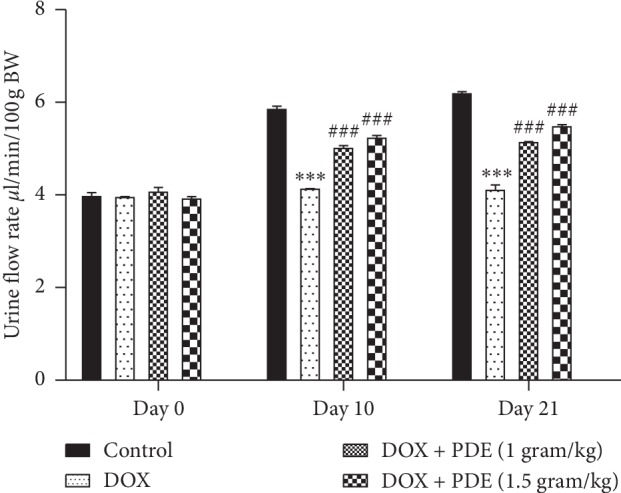
Effects of PDE on urinary flow rate in DOX-induced changes in rats. The values are mean ± SEM (*n* = 6). Statistical analysis was done through two-way analysis of variance (ANOVA) followed by Bonferroni post hoc test. For determination of *p* values, DOX is compared with the control group, and treatment groups were compared with the DOX group. The results are considered significant if *p* < 0.05. ^*∗∗∗*^*p* < 0.001 vs. control and ^###^*p* < 0.001 vs. DOX.

Urine flow rate was reduced in DOX-induced group compared with the control group. This effect showed marked time dependency as urine flow rate almost decreased to half after the 21^st^ day compared with the 10^th^ day. PDE showed slight significant improvement in urine flow rate compared with the DOX group (Figure 3).

#### 3.2.2. Effects of PDE on Urinary Sodium and Potassium Levels

**Figure 4:**
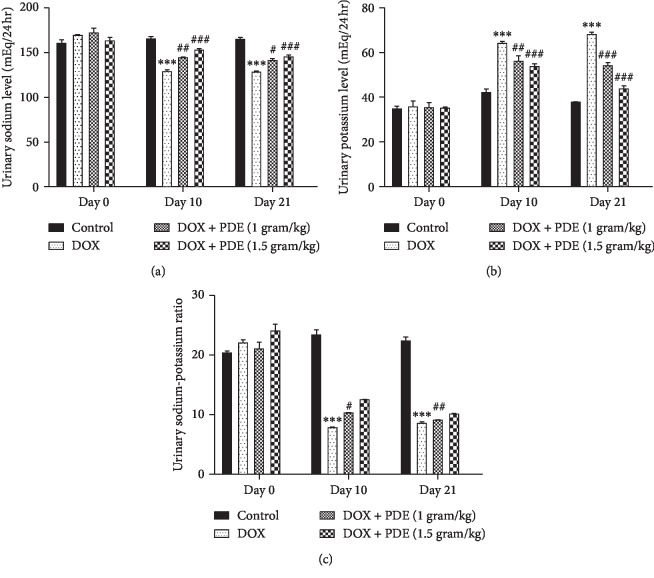
Effects of PDE on urinary (a) sodium, (b) potassium, and (c) sodium-potassium ratio levels in DOX-induced changes in rats. The values are mean ± SEM (*n* = 6). Statistical analysis was done through two-way analysis of variance (ANOVA) followed by Bonferroni post hoc test. For determination of *p* values, DOX is compared with the control group, and treatment groups were compared with the DOX group. The results are considered significant if *p* < 0.05. ^*∗∗∗*^*p* < 0.001 vs. control; ^#^*p* < 0.05; ^##^*p* < 0.01 and ^###^*p* < 0.001 vs. DOX.

Level of urinary sodium decreased in the group treated with DOX at a single dose of 10 mg/kg compared with the normal control group, while there is an increase in the sodium level in groups treated with PDE at a dose of 1 g/kg and 1.5 g/kg (Figure 4(a)). On the contrary, DOX caused a rise in the urine potassium level in group given 10 mg/kg single bolus dose compared with the normal control group. The groups treated with PDE showed a slight decrease in the potassium level given at different doses i.e., at 1 g/kg and 1.5 g/kg (Figure 4(b)). The changes in urinary sodium-to-potassium ratio were measured on three different durations during the study period starting with day 0, continuing on day 10, and finally on day 21. It was observed that DOX-intoxicated group has low levels of urinary sodium to potassium ratio compared with the normal control group (Figure 4(c)). There was significant increase in urine sodium-to-potassium ratio in groups treated with PDE and intoxicated with DOX at doses of 1 g/kg and 1.5 g/kg.

#### 3.2.3. Effects of PDE on Histopathology of Heart and Kidneys

**Figure 5:**
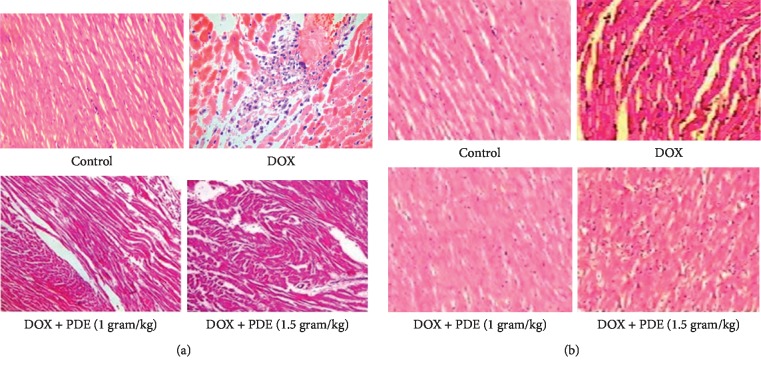
Histopathological analysis of the section of the heart: (a) control (N/S 4 ml/kg); DOX (10 mg/kg); DOX + PDE (1 gram/kg); and DOX + PDE (1.5 gram/kg) in doxorubicin-induced cardiotoxicity. Histopathological analysis of the section of the kidney: (b) control (N/S 4 ml/kg); DOX (10 mg/kg); DOX + PDE (1 gram/kg); and DOX + PDE (1.5 gram/kg) in doxorubicin-induced cardiotoxicity.

Performed histopathological analysis on heart and kidney showed disrupted tissue sections when DOX was administered. Tissue sections showed notable improvement in arrangement with the administration of PDE at the increasing doses of 1 gram and 1.5 grams (Figure 5).

## 4. Discussion

This study was designed to examine the cardioprotective and nephroprotective effects of PDE extract against DOX-induced cardiotoxicity and nephrotoxicity. The intraperitoneal administration of DOX showed cardiomyopathy manifested by raised levels of CK-MB, troponin I, and LDH and are consistent with earlier studies [28]. It has been observed that the mortality rate in animals treated with DOX was 40% compared with the normal control group before the end of experiment which may be due to accumulation of ascites resulting from abnormality in cardiac functioning [29]. Ascites were developed due to extracellular volume expansion and fluid leakage towards interstitium due to tubular disorder and sodium retention as proposed in previous studies [30]. Moreover, nephrotoxicity was also observed by several renal functional parameters which were also a contributing factor in mortality rate. Weight loss was also observed due to less food intake as the toxic effect of drugs affects the intestinal mucosa and decreases appetite by indirect action on the gastrointestinal tract by decreasing the secretion of internal hormones [31]. Improvement in body weight was measured in groups treated with PDE compared with the intoxicated one due to increased food intake and enhancement of intestinal mucosa by use of PDE as it was reported previously [32].

Oxidative stress has been proposed as main stay of DOX-induced cardiomyopathy, and photochemical analysis of PDE showed the presence of flavonids, carotenoids, anthocyanins, procyanidins, phenolic acids, fats, proteins, fibers, and soluble and insoluble form of amino acids [33]. Reduction in levels of CK-MB, troponin I, and LDH levels were observed after administration of the PDE extract at doses of 1 g/kg and 1.5 g/kg, and decreased mortality rate was also observed which suggests cardioprotective action of PDE. Evidence from present study and previous reports support the view that the antioxidant potential of PDE may responsible for the observed cardioprotection.

Plasma sodium level was elevated in rats intoxicated with DOX compared with the normal control, and there was observed a decrease in elevated levels to normal values in groups received PDE as it was also reported previously that elevated levels of sodium was seen in rats having cardiac damage [34]. There was a decrease in plasma potassium level in the group treated with DOX compared with the normal control group as cardiomyopathy caused hypokalaemia [35], and there was increase in potassium level near to normal values when the intoxicated group was treated with PDE and in accordance with previous studies [36].

It was observed that DOX also caused nephrotoxicity due to oxidative stress as free radicals formed and caused tubular atrophy and increased glomerular capillary permeability. Nephrotoxicity was also due to lipid peroxidation and biological macromolecules damage by iron-dependent oxidative damage [37]. Degenerative changes in kidney depend on cumulative dose and duration of treatment as DXR metabolites partly excrete from the kidney. Another mechanism for renal injury is the conversion of DOX to semiquinone free radical by NADPH-cytochrome P-450 which generates hydroxyl radical and superoxide anion which causes lipid peroxidation [38].

It was observed that renal markers like urine output, urine flow rate, urinary sodium level, urinary potassium, and urinary sodium–to-potassium ratio, absolute sodium excretion, and absolute potassium excretion values showed nephrotoxicity caused by DOX. There was a reduction in urine sodium excretion in groups treated with DOX compared with the normal control due to decreased sodium reabsorption due to tubular disorders. This sodium retention was due to stimulation of the cortical collecting duct Na^+^/K^+^ ATPase as it was suggested in previous studies [30].

It was observed that there was an increase in sodium excretion in groups treated with PDE which indicates that the extract has a potential to improve renal functioning as PDE also possess nephroprotective action by rectifying the proximal tubular damage by its antioxidant potential [39]. Urinary potassium level was increased in groups treated with DOX compared with the normal control group due to activation of Na^+^/K^+^ ATPase activity as it was reported in studies conducted in 2000 [30].

In conclusion, coadministrating of PDE extract has a potential to protect against acute DOX-induced cardiotoxicity by normalizing cardiac enzymes, structural abnormalities, and changing the pathways that trigger cardiac apoptosis. Moreover, PDE extract also preserved the structural and functional entities in the renal tissue. On the basis of above findings, it is suggested that the PDE extract owing to its antioxidant potential prevents the DOX-induced cardiotoxicity and nephrotoxicity. However, further studies are needed to evaluate the exact mechanism of action.

## Data Availability

The data used to support the findings of this study are available from the corresponding author upon request.
